# Expression of trypanotolerance in N’Dama x Boran crosses under field challenge in relation to N’Dama genome content

**DOI:** 10.1186/1753-6561-5-S4-S23

**Published:** 2011-06-03

**Authors:** Caleb Orenge, Leonard Munga, Charles Kimwele, Steve Kemp, Abraham Korol, John Gibson, Olivier Hanotte, Morris Soller

**Affiliations:** 1 Kenya Agricultural Research Institute - Trypanosomiasis Research Centre (KARI-TRC), Kikuyu, Kenya; 2 Department of Veterinary Anatomy and Physiology, University of Nairobi, Kenya; 3 School of Biological sciences, University of Liverpool, Liverpool L69 7ZB, UK; 4 International Livestock Research Institute (ILRI), Nairobi-Kenya; 5 Institute of Evolution, University of Haifa, Haifa 31905, Israel; 6 The Centre For Genetic Analysis and Applications, University of New England, Armidale, NSW 235, Australia; 7 School of Biology, University of Nottingham, School of Biology, Nottingham NG7 2RD, UK; 8 Department of Genetics, The Hebrew University of Jerusalem, 91904 Jerusalem, Israel

## Abstract

**Background:**

Animal trypanosomosis in sub-Saharan Africa is a major obstacle to livestock based agriculture. Control relies on drugs with increasing incidence of multiple-drug resistance. A previous mapping experiment in an F2 population derived from the indigenous trypanotolerant N’Dama cattle crossed to susceptible (Kenya)-Boran cattle under controlled challenge, uncovered a number of trypanotolerance QTL (T-QTL). The present study was to determine expression of N’Dama trypanotolerance in a backcross to the Boran under conditions of field challenge, and whether chromosomal regions associated with trypanotolerance in the F2 experiment showed similar effects in the BC population.

**Methods:**

192 backcross animals to the Boran were produced in six batches from June 2001 to December 2006. At one year of age animals were moved to the field and exposed to natural challenge over about one year in Southwest Kenya (Narok). The animals were individually recorded weekly for body weight, packed cell volume, parasitaemia score, and drug treatments, and were genotyped using 35 microsatellite markers spanning 5 chromosomes found in the F2 study to harbour T-QTL.

**Results:**

The F1 were most trypanotolerant, Boran least, and BC intermediate. Females showed distinctly higher trypanotolerance than males. There was a positive correlation in the BC population between trypanotolerance and number of N’Dama origin marker alleles. QTL mapping revealed T-QTL distributed among all five targeted chromosomes, corresponding in part to the results obtained in the F_2_ experiment.

**Conclusions:**

N’Dama origin trypanotolerance is expressed in a BC population under field conditions in proportion to N’Dama origin marker alleles. Consequently, marker assisted selection in such populations may be a means of increasing trypanotolerance, while retaining the desirable productive qualities of the recurrent parent.

## Background

Animal trypanosomosis in sub-Saharan Africa is a major obstacle to livestock based agriculture [1,2]. As a vaccine is not available and vector control measures are ineffective in the long term, control relies on drugs with increasing incidence of multiple-drug resistance [3]. A number of indigenous cattle breeds, of which the West African N’Dama (B. taurus) is the most prominent, are trypanotolerant [4]. However, the N’Dama, although eminently suitable for its present highly extensive agro-ecological niche, is a small animal not suitable for draught purposes, and with limited potential for more intensive meat or milk production. Identifying QTL responsible for trypanotolerance could provide a basis for effective marker-assisted introgression of trypanotolerance from the N’Dama to larger animals more suitable for draught purposes, and with potential for greater productivity under more intensive production conditions. A previous mapping experiment [5] in a N’Dama x Kenya Boran (susceptible B. indicus origin) F_2_ population under controlled challenge, uncovered a number of trypanotolerance QTL (T-QTL). The objective of the present study was to determine whether N’Dama trypanotolerance was expressed in a backcross to the Boran under conditions of field challenge, whether the chromosomal regions associated with trypanotolerance in the F2 experiment showed similar effects in the BC population, and whether trypanotolerance and body weight were closely associated in the BC.

## Methods

192 (N’Dama x Kenya Boran) x Kenya Boran BC animals were produced in six batches from June 2001 to December 2006, using semen of F1 males produced in the previous F2 experiment, and reared in a tsetse fly free area. Some of the batches also included F1 (N’Dama x Kenya Boran) and purebred Kenya Boran animals as controls. At one year of age animals were moved to the field and exposed to natural field challenge by tsetse flies over the course of one year in Southwest Kenya (Narok). All animals were treated when PCV values were below 18% or the animals otherwise showed danger of mortality.

The animals were individually recorded weekly for body weight, packed cell volume, parasitemia score, and drug treatments. From these data a total of 35 trypanotolerance related traits were constructed for each animal. Selected traits are shown in Table 1.For each animal and trait, a standardized trypanotolerance score was calculated, and a composite measure of trypanotolerance was developed based on total standardized trypanotolerance score for all 35 traits. In addition, the BC animals were genotyped using 35 microsatellite markers spanning 5 chromosomes (BTA 2, 4, 7, 16 and 17) found in the F_2_ study to harbour T-QTL. For each BC animal a total N’Dama allele score was calculated as the total number of genotyped markers for which N’Dama alleles were present in the genome. T-QTL mapping for the individual trypanotolerance traits and for total trypanotolerance score was implemented using the Multi-QTL software package (MultiQTL® (http://www.multiQTL.com, and [6]).

**Table 1:** Acronyms (ACR) and definitions of some trypanotolerance traits

ACR	Definition
STR	Total number of positive parasitemia detections over entire challenge period (L).
TPS	Sum of all parasitemic scores over the entire challenge period (L)
NINF	Total number of infection cycles (number of new infections following initial exposure, or treatment) (L)
NIT	Proportion of non-treated parasitemia detections (H)
DF1	No. of days from first exposure to tsetse challenge to first parasitemia detection (H)
DT1	No. of days from first parasitemia detection to *first* treatment for that infection (H)
MPC	Mean PCV of animal during the entire challenge period (H)
WTIT1	Body weight change from initial body weight to weight at first treatment (L)

L, a low value for the trait represents greater trypanotolerance; H, a high value for the trait represents greater trypanotolerance.

## Results

### Gender and genetic type effects

Table 2 shows average BC trait values for the eight selected trypanotolerance traits of Table 1. The average BC animal underwent 3.17 infection cycles (NINF) testing positively for parasitemia in 18.25 tests out of about 52 weekly tests. Infection was rapid, with first parasitemia detection only 14.7 days after exposure, and average of 35.4 weeks from first detection to PCV value of 18% at which time they were treated. During this period animals lost on average 10.06 kg body weight. Over much of the test period the animals were in an anemic state, with average PCV of 24.69% compared to normal PCV in range 32-34%.

**Table 2:** Trypanotolerance effects of gender, genetic type (F1, BC, purebred Kenya Boran) and number of N’Dama origin marker alleles

Trait	BC	Female/ Male	F1/BC	BC/Boran	High/Low
STR	18.25	-0.046 R	-0.466 R***		-0.259 R**
TPS	39.7	-0.044 R	-0.436 R***		-0.132 R**
NINF	3.17	-0.195 R***	-0.334 R***		-0.164 R**
NIT	0.754	0.082 R*	-0.086 S*	-0.635 R**	0.027 R
DF1	14.70	0.161 R	1.075 R***	-0.267 R**	0.210 R
DT1	35.40	0.224 R	0.647 S**	-0.519 R**	1.369 R**
MPC	24.69	0.037 R	0.139 R***	-0.059 S**	0.031 R
WTIT1	-10.06	-0.616 R			-0.427 R**

Average BC trait values; Relative trypanotolerance of female gender compared to male, of F1 compared to BC, of BC compared to Boran, and of High N’Dama allele- number group compared to Low N’Dama allele-number group. R, the first category of the pair is more trypanotolerant; S, the second category of the pair is more trypanotolerant.

Table 2 also shows gender effects as deviation of female BC mean from male BC mean, as a proportion of the BC mean. F1 and Kenya-Boran effects are shown as deviation of their respective means from the BC mean, as a proportion of the BC mean. Also shown is the deviation of the mean of the 30% of BC animals with highest N’Dama allele score from the mean of the 30% of BC animals with the lowest N’Dama allele score as a proportion of the mean trait value of the two groups. Females showed distinctly higher trypanotolerance than males for all eight of the trypanotolerance traits. F1, BC and purebred Kenya-Boran could not be compared with respect to body weight change under infection (WT1T1), since body weight is a major trait difference between the N’Dama and Kenya-Boran, and the animals as yearlings were still in growth phase. Aside from this, F1 animals showed higher trypanotolerance than BC for all traits except NIT (proportion of non-treated parasitemia detections) and DT1 (number of days from first parasitemia detection to first treatment for that infection. Thus, although F1 animals had appreciably fewer parasitemia detections (STR), total parasitemia score (TPS) and infection cycles (NINF) than the BC animals, and took longer until first infection following exposure (DF1), once parasitemia was detected, they progressed more rapidly to treatment, but overall maintained PCV better across the entire challenge period. The purebred Kenya Boran animals had to be removed from the challenge situation well before the end of the test period, and hence could not be compared to the BC for number of parasitemia detections (STR) sum of parasitemia scores (TPS) or number of infection cycles (NINF). However, they were infected sooner (DF1) and progressed to treatment more rapidly (DT1) than the BC animals.

### Marker associated effects

As shown in Table 2, the 30% of BC animals with the highest number of N’Dama origin alleles at the 35 monitored markers were more trypanotolerant for all eight trypanotolerance traits, than the 30% of BC animals with the lowest number of N’Dama origin alleles at these markers. This is a remarkable results considering that only markers on five of the 29 autosomal chromosomes were monitored. QTL mapping revealed T-QTL distributed among all five targeted chromosomes, corresponding in part to the results obtained in the F_2_ experiment.

### Body weight and trypanotolerance

Body weight of the BC animals prior to challenge (148.7 kg) was only slightly less than purebred Boran (153.0 kg), indicating that the BC animals retained at least some of the superior production features of the Boran. Body weight was not correlated to total trypanotolerance score or with total number of N’Dama alleles at the monitored markers.

## Discussion

The results of this study show that trypanotolerance of N’Dama origin comes to expression in a BC population under field challenge, indicating that an appreciable component of the tolerance is co-dominant and general in nature. Trypanotolerance was in proportion to overall proportion of N’Dama genome, with F1, BC and purebred Kenya-Boran ranking in that order, and also in direct proportion to the number of N’Dama alleles at the monitored marker loci. The large effects associated with the total number of N’Dama marker alleles supports the F2 mapping results that led to the choice of these five chromosomes as having particular association with trypanotolerance. It was encouraging that body weight of the BC animals was only slightly less than that of the purebred Boran, and was not tightly associated with either total trypanotolerance score or total number of N’Dama origin marker alleles, indicating that trypanotolerance is not inimical to larger body size.

## Conclusions

This demonstrates that trypanotolerance observed under highly controlled conditions is also effective under field challenge. Also, that trypanotolerance is positively associated with number of N’Dama origin alleles, is expressed in heterozygous state, and does not appear to be tightly correlated with body weight. This suggests that MAS or genome-wide selection for trypanotolerance and mass selection for body weight could be used to increase both trypanotolerance and body weight in appropriate crosses. BC populations to the more productive breed would be a useful starting point for such breeding programs.

## Competing interests

None of the co-authors has competing interests.

## Authors' contributions

CO carried out all genotyping and statistical and QTL mapping analyses, LM did the phenotyping, CK provided some guidance in the initial genotyping process (DNA extraction and quantification), AK directed the QTL mapping, JG, O.H., AK and MS designed the study and provided project guidance and supervision. SK provided additional project guidance. MS participated in statistical analyses, presented the study at the AGAH meeting in Paris, and wrote the article for BMC proceedings.
